# Back to the Future, Current Trends in Breakthrough Pain Treatments

**DOI:** 10.4103/0973-1075.76237

**Published:** 2011-01

**Authors:** Max Watson

**Affiliations:** Visiting Professor Palliative Medicine, University of Ulster; Consultant Palliative Medicine Northern Ireland Hospice, Belfast and Princess Alice Hospice, Esher; Special Adviser the Hospice Friendly Hospitals Programme, Dublin,

**Keywords:** Breakthrough pain, Cost benefit ratio, Background analgesia

## Abstract

The importance of effectively managing breakthrough pain for patients on long term background analgesia has recently lead to the development of a range of new products aimed at filling this need. This review article looks at the reasons behind these developments and their implications for clinical practice in resource limited situations.

Over the past few years in the UK the major emphasis in new preparations to tackle palliative pain has continued to focus on improving the care of patients with breakthrough pain.

The term breakthrough pain typically refers to a transitory flare of pain in the setting of chronic pain managed with opioid drugs.[1]

It is usually related to background pain and is typically of rapid onset, severe in intensity, and generally self-limiting with an average duration of 30 min.[2]

Three types of breakthrough pain are described:

1. Incident pain: commonest and related to movement/coughing
2. Idiopathic/ spontaneous pain: no identifiable cause; lasts longer than incident pain
3. End of dose failure: prior to scheduled dose of analgesia; gradual onset. Often not regarded as true breakthrough pain.[3]

## MANAGEMENT

Portenoy suggested three principles for management: implementing primary therapies (surgery, radiotherapy, chemotherapy), optimizing around-the-clock medication, and specific pharmacological interventions.[1]

## CURRENT GUIDELINES

Currently there is little to guide the physician looking after a patient with breakthrough pain who is only taking paracetamol, an NSAID, a weak opioid, or any combination of these.

Traditionally, a breakthrough dose of oral morphine of one-sixth of the total 24-h dose (equivalent to the four-hourly dose) has been used.[4] However, many centers now advocate using 10% of the total daily regular dose as the breakthrough dose on the grounds that it provides a better balance between top-up analgesia and adverse effects.[5,6]

Immediate-release fentanyl products should probably only be started under the supervision of a specialist. Although they have a faster onset of action than immediate-release morphine they are not bioequivalent, the dose must be individually titrated. Serious adverse effects and deaths have been reported when they are incorrectly used.[5]

Breakthrough analgesia for incident pain requires a lot of cooperation, coordination, and planning in order to be successful.

The goal of the ideal breakthrough preparation has led several companies independently producing their own product aimed at delivering a dose of Fentanyl in a formulation which has a rapid onset of action and a reduced hangover effect.

Thus we now have buccal, sub-lingual and intra-nasal preparations of Fentanyl. That so much time, effort and expense has gone into the development of such products is an indication of how large the drug companies feel that the potential breakthrough medication market is. Complaints upheld by the pharmaceutical watchdog in the UK against two of the most widely marketed products for sharp practice, is another indicator as to how valuable this product niche is deemed.

Dr Andrew Wilcock, Reader in Palliative Care in Nottingham and editor of the Palliative Care Formulary, compared the research basis on which the new preparations have been based.

## SUMMARY OF THE CLINICAL STUDIES

1. None of the new fentanyl preparations have been robustly investigated with an appropriate active comparator such as oral morphine.
2. All demonstrated significant and dose-related responses compared with placebo in patients with cancer-associated pain.
3. All studies appear to have used an enhanced patient recruitment process which may have distorted the efficacy results in favor of the new treatment.
4. All products appear to produce rapid analgesia in a substantial proportion of patients. There is a lack of evidence directly comparing fentanyl products for breakthrough cancer pain.
5. All of the immediate-release fentanyl preparations provide adequate pain relief within 10-15 min, when compared to placebo [Table 1].

**Table 1:** Pharmacokinetic/dynamic profiles of different products

Pharmacokinetics (1-4)	UK Proprietary Name	Absorption	Bioavailability	Mean maximal plasma concentration	Time to pain relief post dose
	Abstral	Rapid absorption over about 3 minutes.	~70%	0.2-1.3ng/mL (after taking 100 - 800mcg) reached within 22.5 - 240mins.	10 minutes
	Effentora	50% of the dose absorbed transmucosally,50% swallowed & absorbed slowly from the GIT	65%	0.6 to 1.44ng/ nL are reached in 46.8 minutes (range 20-240).	10 minutes
	Instanyl	Absorbed rapidly through the nasal mucosa.	89%	0.35-1.2ng/mL reached within 12- 15 minutes from 50-200mcg doses.	10 minutes (range 7-11 minutes)

Switching from one product to another must not be done at a 1:1 ratio due to differences in bioavailability and the absorption profiles: a new dose titration must be carried out. This may result in insufficient pain control during the titration phase

## SUMMARY OF THE SAFETY DATA

1. In general the fentanyl products were well tolerated.
2. The majority of adverse effects were to be expected with the use of a potent opioid generally, or fentanyl more specifically.
3. The wide range of fentanyl products can lead to errors in dosing due to differences in pharmacokinetic/dynamic profiles.
4. The products are not interchangeable.
5. There is potential for prescribing and dispensing errors if more than one formulation is available locally.

### Titration

Baseline maintenance opioid use is an absolute requirement before starting one of these products, i.e.

At least 60 mg of oral morphine daily, 25 micrograms of transdermal fentanyl per h, 30 mg of oxycodone daily, or 8 mg of oral hydromorphone daily or an equianalgesic dose of another opioid for a week or longer.

### Cost implications

**Figure 1:**
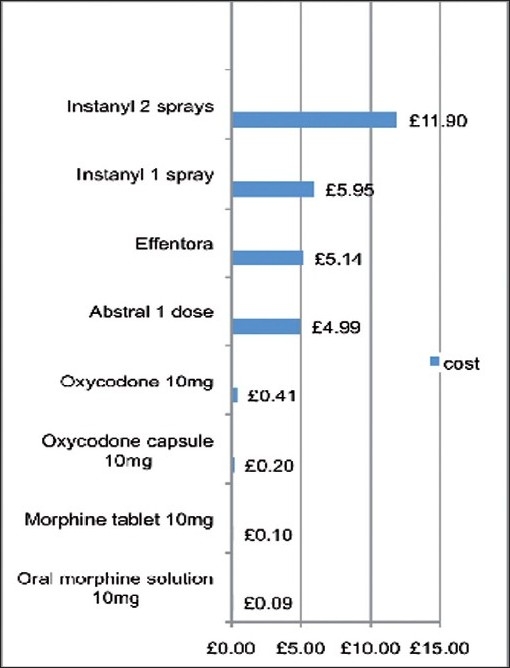
Relative pricing of rapid-onset fentanyl products, UK Prices

The review of rapid-onset fentanyl products highlights a fundamental scenario which confronts physicians seeking to provide optimal care for patients in a resource-limited environment [Figure 1].

The scenario is this. A physician has to choose between two preparations. One has been used for many years and has well-known drawbacks and benefits. It costs 10 paisa per use. A newer preparation is created. It is equally effective in terms of managing pain but has reported fewer side-effects. It costs 60 rupees a dose. Being a newer preparation, its full range of benefits and side-effects has potentially yet to be discovered.

How can the physician evaluate for the individual patient which drug is most appropriate? Making the choice involves a range of different factors only one of which is the fact that the newer preparation is 600 times more expensive than the old. These factors include:

1. Drug availability
2. Patient need, expectation and choice
3. Cost
4. Physician experience of using the preparation appropriately
5. Patient choice
6. Capacity of thepatient and patient’s family to deal with the drug administration regime
7. New drug phobia or new drug philia which can often be aggravated by aggresive promotion and advertising

Clearly, a new drug is not automatically better than an old drug. Equally, not all old drugs are better for individual patients than new drugs. That said there is a multi-million rupee industry which has to convince us that the investment in the creation of new preparations has been well spent.

Such a commercial drive encourages physicians to either feel guilty if they do not choose the latest and most expensive and supposedly “best” option for their patients or promotes a smaller minority to aggressively resist any change from the older preparations.

In our fast-changing world with ever-increasing patient expectation the importance of the doctor/patient relationship cannot be overemphasized. It is only in the context of this relationship that the best pharmacological decisions can be made.

In short, no new preparation, no matter what it claims, can ever replace the wisdom of a skilled physician making an appropriate assessment and selection of appropriate treatment to help meet the individual need of the particular patient.

Even in the business of the most pressurized clinic it is this skill which remains the most durable of therapeutic interventions for breakthrough pain.
